# Deciphering Microbial Communities and Distinct Metabolic Pathways in the Tangyin Hydrothermal Fields of Okinawa Trough through Metagenomic and Genomic Analyses

**DOI:** 10.3390/microorganisms12030517

**Published:** 2024-03-04

**Authors:** Jiake Li, Haojin Cheng, Fu Yin, Jiwen Liu, Xiao-Hua Zhang, Min Yu

**Affiliations:** 1 Frontiers Science Center for Deep Ocean Multispheres and Earth System, College of Marine Life Sciences, Ocean University of China, Qingdao 266003, China; lijiakee@outlook.com (J.L.); chenghaojin@stu.ouc.edu.cn (H.C.); yinfuhaida@163.com (F.Y.); liujiwen@ouc.edu.cn (J.L.); xhzhang@ouc.edu.cn (X.-H.Z.); 2 Key Laboratory of Evolution & Marine Biodiversity (Ministry of Education), Institute of Evolution & Marine Biodiversity, Ocean University of China, Qingdao 266003, China; 3 Laboratory for Marine Ecology and Environmental Science, Laoshan Laboratory, Qingdao 266071, China

**Keywords:** Tangyin hydrothermal vent, microbial communities, metagenomics, metagenome-assembled genomes

## Abstract

Deep-sea hydrothermal vents have been extensively explored around the globe in the past decades, and the diversity of microbial communities and their ecological functions related to hydrothermal vents have become hotspots in the study of microbial biogeochemistry. However, knowledge of dominant microbial communities and their unique metabolic characteristics adapting to hydrothermal vents is still limited. In our study, the sediment sample near the Tangyin hydrothermal vent in the southern part of the Okinawa Trough was collected, and the most abundant phyla are Proteobacteria and Desulfobacterota based on the 16S rRNA genes and metagenome sequencing. Metagenomic analysis revealed that methane metabolism, sulfur reduction, and Fe^2+^ uptake were abundantly distributed in hydrothermal sediment. In addition, most of the metagenomic assembly genomes (MAGs), belonging to *Chloroflexota*, *Desulfobacterota*, and *Gammaproteobacteria*, were found to be involved in methanogenesis, sulfur oxidation/reduction, and ferrous/ferric iron metabolisms. Among these MAGs, the two representative groups (*Bathyarchaeia* and *Thioglobaceae*) also showed distinct metabolic characteristics related to carbon, sulfur, and iron to adapt to hydrothermal environments. Our results reveal the dominant microbial populations and their metabolic features in the sediment near the Tangyin hydrothermal fields, providing a better understanding of microbial survival strategies in the extreme environment.

## 1. Introduction

Deep-sea hydrothermal vent systems are characterized as some of the most distinct and complex marine environment systems, with reducing fluids mixing with oxic deep-sea waters and forming steep physical and geochemical gradients [1]. Since the hydrothermal vent was discovered in 1977 [2], microbial communities in different ecological niches associated with hydrothermal vents have been widely explored and well-investigated, including hydrothermal fluids, plumes, chimneys, and sediments [1,3]. The chemical and thermal gradients provide a wide range of niches for microbial communities living there, which could be considered ideal fields for studying microbial community structures and their response to hydrothermal environments [3,4]. Using high-throughput sequencing, diverse bacterial and archaeal populations have been found in different hydrothermal fields, and the physical and chemical gradients, as well as geologic settings, are the main environmental factors that have shaped the microbial community structure [5,6].

Hydrothermal vents are some predominant sources of sulfur elements in the ocean and play a crucial role in global sulfur cycling. Chemotrophic microorganisms, such as *Sulfurospirillum*, *Sulfurovum*, *Sulfurimonas*, and *Thiomicrospira*, are recognized as key producers and abundant bacteria in hydrothermal fields [7,8]. These free-living or symbiotic bacteria use reduced sulfur, H_2,_ and CH_4_ as electron donors to fix carbon, thereby supplying carbon and energy resources to other organisms inhabiting this marine environment [9]. In addition to sulfur oxidation, recent research has also focused on sulfate-reducing bacteria (SRB), which play crucial roles in sulfur and carbon cycling in hydrothermal fields [10]. However, their ecological roles are still poorly understood. With the advancement of sampling and sequencing techniques, the number of bacteria strains involved in sulfur oxidation and sulfate reduction has been isolated, and their genetic and metabolic characteristics have been thoroughly investigated [1,11,12]. It has been found that sulfur-oxidizing bacteria (SOB) and SRB were both abundant and linked to different carbon metabolic pathways in two low-temperature hydrothermal chimneys on the Southwest Indian Ridge [13,14]. SRB inhabiting the inner chimney were found to enrich genes involved in short-chain alkane oxidization and hydrogen utilization, which suggests that SRB also play a significant role in energy-source cycling. Further efforts in studying the distribution and metabolic pathways of SRB in hydrothermal fields are needed to elucidate their ecological function in sulfur and carbon cycling.

The Okinawa Trough is a back-arc basin located along the eastern margin of the Eurasian continent, extending for more than 1200 km, and characterized by both hemipelagic and volcanic sediments [15]. It is divided into three parts based on water depths and gap locations, i.e., the northern, middle, and southern parts. It encompasses sediments enriched in H_2_, CO_2_, CH_4_, NH_4,_ and H_2_S, which are derived from sedimentary organic matter and magmatic gases [16,17]. Until now, many hydrothermal fields have been identified in the Okinawa Trough [7]. Among these fields, the “Tangyin” hydrothermal vents, were found at a depth of 1206 m in the southern Okinawa Trough [18]. Sulfide mineral particles such as pyrite, chalcopyrite, galena, and methane (~4.1 μmol/L) have been observed in this hydrothermal vent. It is documented that the in situ geochemistry and temperature (up to 300 °C) of the sediments promote the thermal decomposition of organic matter into CO_2_, and the biogenic production of CH_4_ [19]. For example, the thermal decomposition of organic matter is a key factor that can influence the concentration of CO_2_ in hydrothermal fluids, whereas CH_4_ was found to be primarily microbial, originating in the pore water [17]. The geochemical characteristics of the Okinawa Trough also significantly impact the microbial population, and many studies have revealed the relationship between microbial community structures and environmental factors, primarily in the middle Okinawa Trough [3,20,21,22,23]. However, the ecological functions of the bacteria inhabiting the hydrothermal sediment in the southern Okinawa Trough were poorly understood.

In this study, we collected one (up to 20 cm) sediment from the Tangyin hydrothermal site in the southern Okinawa Trough for 16S rRNA and metagenomic-assembled genome (MAG) analyses. Functional and taxonomic annotations of genes from the MAGs were analyzed to identify the dominant bacterial populations and their potential roles in these hydrothermal fields. Representatives of the MAGs were compared with phylogenetically related microorganisms from other environments to gain a better understanding of their unique metabolic characteristics and roles in carbon, sulfur, and iron cycles in the sediment of the Tangyin hydrothermal field.

## 2. Materials and Methods

### 2.1. Sampling Site and DNA and RNA Extraction

**Figure 1:**
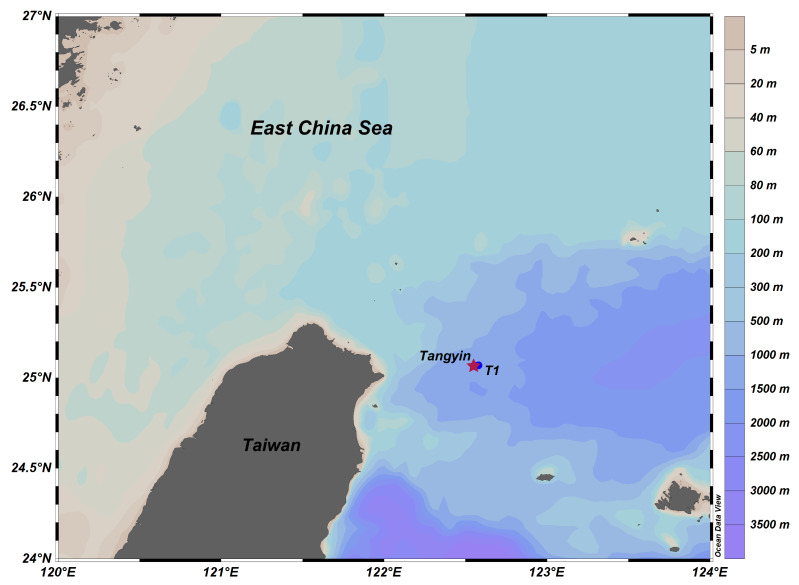
Overview of the sampling site and Tangyin hydrothermal field at the southern Okinawa Trough. The red star represents the vent position.

One sediment (up to 20 cm) sample (T1, 122°34.70′ E, 25°4.24′ N), which was 1.2 km from the Tangyin hydrothermal vent (122°34′ E, 25°4′ N), was obtained onboard using a box corer deployed from the R/V Kexue no. 1 in May 2014 (Figure 1). Upon collection, the sediment was divided into three replicates, frozen immediately in liquid nitrogen, and stored at −80 °C until DNA/RNA extraction. In brief, for the 16S rRNA genes analyses, 0.5 g of sediment was extracted using the DNeasy PowerSoil Pro Kit following the manufacturer’s suggestion. To analyze active microbial communities, we also extracted the environmental RNA using the Direct-zol RNA Miniprep kit, and DNA removal was performed using a TURBO DNA-free kit. Then the reverse transcription of RNA using the SuperScript III First-Strand Synthesis System was performed. Finally, we obtained the cDNA for sequencing. To obtain better quality DNA for the metagenomic analysis, we extracted 10 g of sediment using a DNA extraction protocol that involved osmotic lysis of the cells, followed by 1% sodium hexadecyltrimethylammonium bromide (1% CTAB), 15% dodecyl sulfate (15% SDS), and proteinase K (50 μg/mL) treatments to remove humic acids and proteins from the crude DNA extract [24]. The crude DNA was purified using spin columns and was subsequently extracted from the 0.5% low melting point agarose gel. The quality of DNA was assessed spectrophotometrically with Nanodrop.

### 2.2. DNA and RNA Based on 16S rRNA Gene Amplicon Sequencing

The DNA and cDNA samples were sequenced in triplicate by Majorbio Bio-Pharm Technology Co., Ltd. (Shanghai, China). The V4 regions of the 16S rRNA genes/transcripts were amplified using the primer pairs 515F and 806R [25] and then sequenced by Illumina MiSeq PE300 (MiSeq Reagent Kit v3) platform (Wuhan, China). The bioinformatics analysis toolkit QIIME2 was used to filter the raw data [26]. The raw reads were trimmed with FASTP to remove low-quality (<20 bp) and short-length sequences (<100 bp). The paired-end DNA sequences with at least a 10 bp overlap and <5% mismatches were joined using FLASH (v1.2.11) [27]. Moreover, chimeric sequences, barcode sequences, and primers were removed using the DADA2 plugin in the QIIME2 software (v2020.11). Then the sequences of total and active bacteria and archaea were clustered into amplicon operational taxonomic units (OTUs) at a 97% sequence similarity level. Taxonomic assignments were annotated with the SILVA databases (v138) [28].

### 2.3. Gene Functional Classification of the Metagenome

The raw reads of the metagenomes that contained >10% undefined bases, >40% low-quality bases (quality score < 20), and >15 bases matching the adapters were removed [29]. The assembly of contigs was performed using MetaWRAP metaSPAdes, and gene prediction was performed by prodigal (v2.6.3) with default parameters [30]. High-quality gene catalogs were constructed from encoding genes after de-redundancy with a threshold of 95% sequence similarity by CD-HIT (v4.6.8), and sequences of <100 bp were discarded [31]. All the predicted genes were searched against KEGG databases for functional characterization. The methods for calculating the gene abundance were according to Xue et al. [32]. Briefly, the non-redundant gene catalogs were mapped against the clean reads by BWA-MEM (v0.7.17) using default parameters [33], and the average coverage of each gene was determined by BBMap (v38.90) [34]. The functional assignment of each gene was performed by Diamond (v2.1.8) using the BLASTp search with the e-value threshold of 1 × 10^−10^ against the KEGG database (v89), and only the best hit was retained [35]. The taxonomic assignment of each gene was performed with MEGAN (v6.21.7) [36].

### 2.4. Metagenomic Binning, Taxonomic Assignment, and Functional Characterization

Metagenomic binning was performed using MetaWRAP [37] with initial assembly using MetaWRAP metaSPAdes. Completeness and contamination of metagenomic assembly genomes (MAGs) were assessed using CheckM [38], and MAGs with completeness above 50% and contamination lower than 10% were considered for further analysis. The taxonomy of MAGs was annotated by using GTDB-Tk (v2.3.0) [39]. Phylogenomic trees of these genomes and reference genomes were constructed based on the single-copy, protein-coding marker genes detected by OrthoFinder (parameters: -t 36 -a 36 -S diamond -M msa -A mafft -T iqtree) [40]. Briefly, those single-copy sequences were aligned with MAFFT (v7) [41], trimmed with trimAl (v1.2) [42], and then submitted to IQ-TREE (v1.6.1) [43] for tree construction. The relative abundance of each MAG was calculated using Anvi’o (v7.1) and MicrobeCensus (v1.1.1) [44] and normalized using the parameter reads per kilobase million (RPKM). Genes in each MAG were predicted using Prokka (v2.6.3) and all the genes were searched against KEGG databases for functional characterization. The completeness of metabolic pathways was determined using the KEGG decoder [45].

## 3. Results

### 3.1. Taxonomic Compositions by the 16S rRNA Gene and Metagenomic Gene

**Figure 2:**
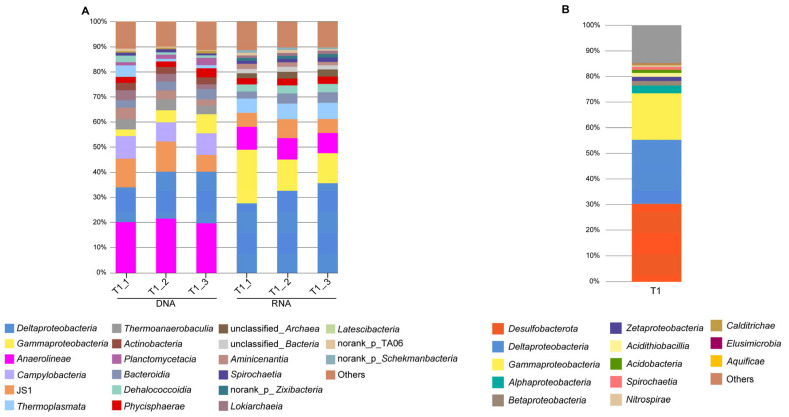
Microbial community composition based on (**A**) 16S rRNA gene/transcripts and (**B**) metagenomic genes from the sediments near the Tangyin hydrothermal field at phylum/class levels.

Average totals of 17,191,187 high-quality 16S rRNA genes and 15,238,686 transcript sequences were obtained from the sediment sample. Totals of 2449 and 3413 operational taxonomic units (OTUs) were classified based on a 97% sequence similarity, respectively. The microbial community in Tangyin hydrothermal vents was mainly composed of Proteobacteria, *Anaerolineae*, *Campylobacteria*, and JS1 in DNA samples. *Proteobacteria* is mainly composed of *Delta*- and *Gammaproteobacteria* (Figure 2A). Our RNA results confirmed the prokaryotic composition identified with our 16S rRNA gene analyses, showing more abundant *Delta*- and *Gammaproteobacteria* and less *Anaerolineae* and JS1 compared to the DNA result.

As for metagenome, a total of 24,354,897 reads were obtained. After the taxonomic assignment of each gene was performed with MEGAN, bacteria belonging to *Desulfobacterota* (30%), *Deltaproteobacteria* (24.3%), and *Gammaproteobacteria* (18.1%) were absolutely dominant (Figure 2B).

### 3.2. Genes Involved in Carbon, Sulfur, and Iron Metabolisms

**Figure 3:**
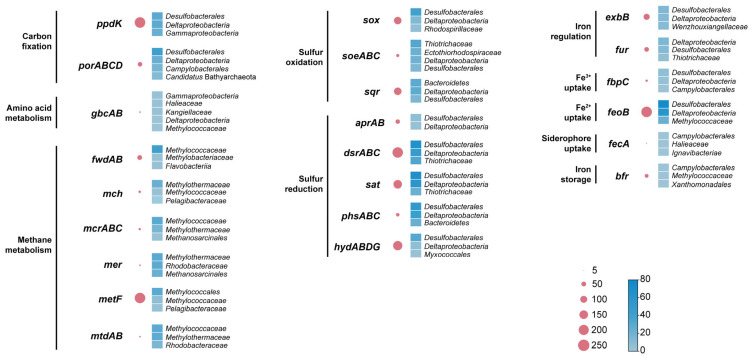
The abundance of genes associated with carbon, sulfur, and iron metabolisms in the metagenome. The bubble plot shows the abundance of genes (RPKM), and the heatmap shows the community composition of these genes.

The functional classification of non-redundant genes using KEGG databases showed that genes involved in energy production, such as carbohydrate, amino acid, and methane metabolisms, were the most abundant (Figure 3). Most of these genes could be classified as belonging to *Gammaproteobacteria*, *Deltaproteobacteria*, and *Desulfobacterales*. Notably, many genes were involved in methane metabolism, and methylene tetrahydrofolate reductase (MetF) was the most abundant enzyme in the metagenomic analyses. MetF catalyzes the production/oxidation of methane, which is the key enzyme of hydrogenotrophic methanogenesis and the anaerobic oxidation of methane. In addition, the related enzymes also included tungsten, containing formylmethanofuran dehydrogenase (FwdAB), methenyltetrahydromethanopterin cyclohydrolase (Mch), methylenetetrahydromethanopterin reductase (Mer), and methylenetetrahydromethanopterin dehydrogenas (MtdAB) [46,47,48]. Genes encoding MetF were identified mainly in *Methylococcales* and *Methylococcaceae* MAGs. However, the representative enzymes for methane production/oxidation, such as McrABC, were rarely found in this study.

Genes involved in sulfur oxidation were reported to participate in a thiosulfate-oxidizing multienzyme system (SOX system), including SoxXYZABDEFGH [49]. We identified SOX genes (*soxB*, *soxD*, *soxYZ*) that showed high relative abundances. Similarly, enzymes involved in assimilatory and dissimilatory sulfate reduction were enriched, including the sulfate adenylyltransferase (Sat), adenylyl sulfate reductase subunits A and B (AprAB), and sulfate reductase subunits A, B, and C (DsrABC). Bacteria belonging to the class *Desulfobacterales* showed high relative abundances of SOX and dissimilatory and assimilatory sulfate reduction genes. Overall, *Deltaproteobacteria* was the main taxon encoding for dissimilatory and assimilatory sulfate reduction genes.

Microorganisms in hydrothermal fields employ different strategies to acquire inorganic and organic iron, such as ferrous iron uptake (Feo) systems, ABC-type transporters [50], and TonB-dependent transporters [51]. Among the Fe-associated genes, we found genes associated with transporting (*fbpC*), uptake (*fecA*), and transcriptional regulation (*fur*) of the Fe^3+^ [52,53] uptake of Fe^2+^ (*feoB*) [54], and genes related to iron storage (*bfr*) [54] and the uptake of siderophores (*exbB*) [55]. Many of these genes were related to Fe^3+^ transport/uptake and were primarily affiliated with *Deltaproteobacteria* and *Desulfobacterales*, with a notable dominance of *Desulfobacteraceae*. Despite the relatively low abundance of bacteria originating from *Campylobacterales* in the metagenomic analysis, genes involved in the uptake of Fe^3+^ and iron storage were predominantly identified within this taxon.

### 3.3. Taxonomic Assignments and Carbon, Sulfur, and Iron Metabolisms of MAGs

**Figure 4:**
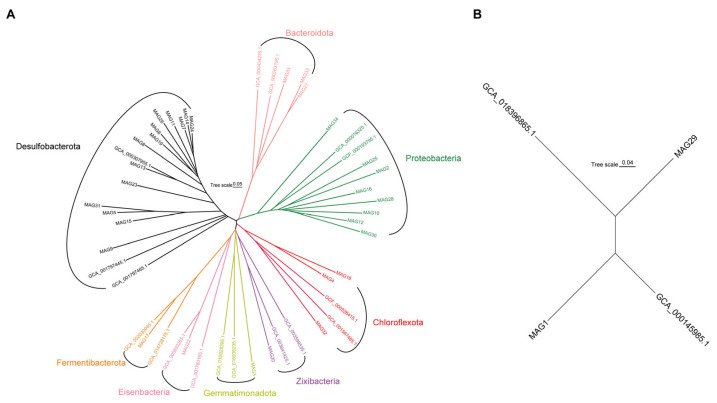
Phylogenetic inference of 32 bacterial MAGs (**A**), 2 archaeal MAGs (**B**), and 19 corresponding reference genomes based on 120 and 122 single-copy and protein-coding marker genes, respectively (Table S1).

We recovered 34 MAGs (Figure 4) affiliated with 8 prokaryotic phyla. The dominant phyla included Desulfobacterota, Proteobacteria, Bacteroidota, and Chloroflexi (Figure 4). *Desulfobacterales* and *Desulfobulbales* were identified as the primary SRB and only the family *Thioglobaceae* was identified as the dominant SOB. Furthermore, the archaeal MAG1 and MAG29 were classified as *Bathyarchaeota* in the phylum *Thermoproteota*.

**Figure 5:**
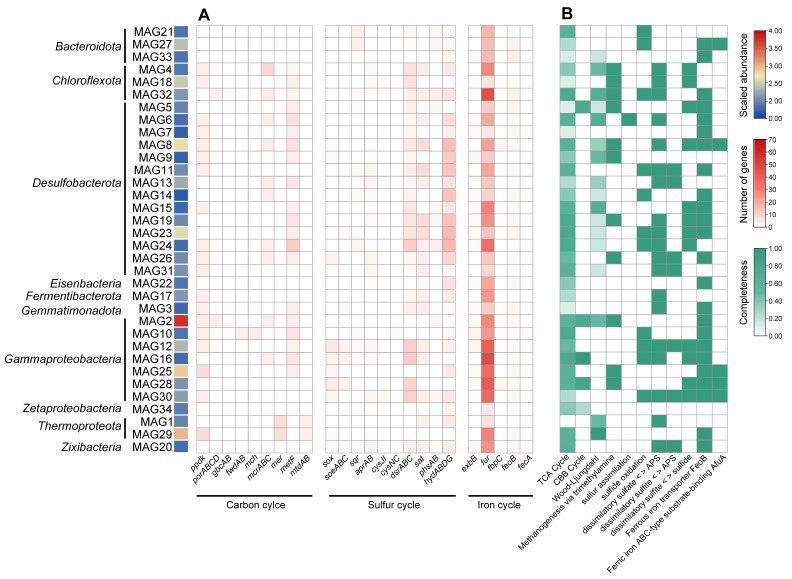
A taxonomic and genetic overview of MAGs associated with carbon, sulfur, and iron cycles in the sediment of Tangyin hydrothermal fields. (**A**) Distribution of functional genes related to carbon, sulfur, and iron cycles in MAGs. The shade of the color represents the number of genes in MAGs. (**B**) Completeness of metabolic pathways related to carbon, sulfur, and iron cycles in MAGs.

Genes in each MAG were annotated according to the KEGG database, and their functions were further analyzed. Twenty-nine MAGs associated with *Desulfobacterota* and *Gammaproteobacteria* contained carbon fixation-associated genes. However, there are fewer MAGs (containing *gbcAB* genes) involved in amino acid metabolism. Interestingly, we found that more MAGs were involved in methane metabolism, containing *mcrABC* and *metF* genes, which were predominantly found in *Chloroflexota*, *Desulfobacterota*, *Gammaproteobacteria*, and *Thermoproteota*. In addition, the complete trimethylamine pathway for methyl-metabolism was identified in *Chloroflexota*, *Desulfobacterota*, and *Gammaproteobacteria*, and the acetate pathway was found in *Thermoproteota* (Figure 5).

Thirty-three MAGs had genes associated with sulfate reduction, which were affiliated with 9 phyla (Figure 5B). The MAGs associated with sulfur metabolism primarily consisted of *Desulfobacterota*, *Gammaproteobacteria*, *Bacteroidota*, and *Chloroflexota*. The result was consistent with the high relative abundance of assimilatory and dissimilatory sulfur reduction genes. Furthermore, we also identified 17 sulfur oxidation-associated MAGs and almost all of them belonged to *Gammaproteobacteria* (Figure 5A).

The taxonomic and genetic overview showed that two genes associated with iron transporting (ferrous iron transporter FeoB and ferric iron ABC-type substrate-binding protein AfuA) were annotated in these MAGs. Many of these MAGs were related to transcriptional regulation (*fur*) and Fe^2+^ transport/uptake (Ferrous iron transporter FeoB) and were primarily affiliated with *Zixibacteria*, *Chloroflexota*, Thermoproteota, and *Desulfobacterota*, with a notable dominance of *Gammaproteobacteria*. However, the number of MAGs containing the gene *feoB* was relatively low, and it differed from the result of non-redundant genes (Figure 5A).

### 3.4. The Unique Metabolic Features of Representative MAGs

**Figure 6:**
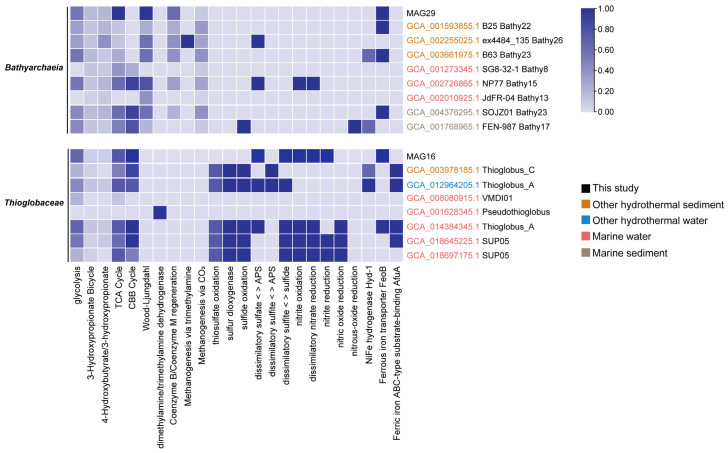
Comparison of metabolic pathways of representative MAGs with reference genomes. The completeness of pathways related to carbon, sulfur, and iron metabolisms is displayed in the heatmap. MAGs in this study are colored in black, and reference genomes from different marine environments are distinguished by different colors.

Among these 34 MAGs, MAG29 and MAG16, which belonged to *Bathyarchaeia* and *Thioglobaceae*, were selected and compared with reference genomes to elucidate their roles in the Tangyin hydrothermal setting. Microbes in these two groups were reported to play important roles in sediments and are widely involved in carbon, sulfur, and iron metabolisms, but their metabolic characteristics and adaptive strategies in hydrothermal vents were poorly understood. To explore their metabolic potential in the Tangyin vent, 8 reference *Bathyarchaeia* genomes (different groups: Bathy8, Bathy13, Bathy15, Bathy17, Bathy22, Bathy23, and Bathy26) [51] and 7 reference genomes of *Thioglobaceae* (*Pseudothioglobus*, *Thioglobus*, VMDI01 and SUP05) from various marine environments were downloaded from GTDB for comparison (Figure 6, Table S2).

We found that archaea belonging to *Bathyarchaeia* in other marine environments contained genes associated with the Calvin–Benson–Bassham (CBB) cycle for carbon fixation. In contrast, MAG29 in this study was only found in the complete Wood–Ljungdahl pathway. In addition, the 3-hydroxypropionate/4-hydroxybutyrate pathway was found in each of these genomes, although they were incomplete. The other *Bathyarchaeia* genomes from the group Bathy26 could be involved in methyl metabolism via trimethylamine. In addition, MAG29 has genes encoding for FeoB, which is responsible for the Fe^2+^ uptake. This feature was mainly observed in *Bathyarchaeia* genomes from sediment samples. From MAG 29 we did not recover genes related to sulfur oxidation, sulfate, and nitrate reduction pathways, which might be attributed to its incomplete genome and requires further investigation.

MAG16 in our study exhibited more capabilities involved in carbon, sulfur, and iron metabolisms. Similar to genomes from other marine environments, the CBB cycle was found to be the primary pathway in MAG 16 for fixing carbon. We also found a dissimilatory sulfate or sulfite reduction pathway in MAG16, and related genes here may be involved in sulfur oxidation, but genes related to the oxidation of different sulfur-containing compounds were absent. However, these genes involved in sulfur oxidation were commonly found in *Thioglobaceae* genomes from other marine environments. In addition, MAG16 contained genes related to nitrite oxidation, dissimilatory nitrate reduction, and nitrite reduction, whereas these genes were absent in the genome from other hydrothermal sediment. As for iron metabolism, MAG16 only had ferrous iron transporter FeoB, but the reference genome from other hydrothermal sediment had NiFe hydrogenase Hyd-1 and ferric iron ABC-type substrate-binding AfuA.

## 4. Discussion

Previous studies suggested that there were plenty of uncultured and novel bacteria in the hydrothermal field [11]. The community structures of bacteria and archaea in various hydrothermal fields of the Okinawa Trough, particularly Yonaguni Knoll IV and Iheya North Knoll hydrothermal fields, have been extensively studied using culture-dependent methods, 16S rRNA gene amplicon sequencing, and metagenomic sequencing [21,23,52,53,54,55]. However, microorganisms showed diverse community compositions and functions because of the different geochemical characteristics, which formed the different physical and chemical gradients in the hydrothermal fields. For example, the dominant bacterial and archaeal groups inhabiting deep-sea sediments overlying a natural CO_2_ lake were found to be anaerobic methanotrophic archaea ANME-2c, the Eel-2 group of *Deltaproteobacteria* and sulfur-metabolizing chemolithotrophs within the *Gamma*- and *Epsilonproteobacteria* at the Yonaguni Knoll IV hydrothermal field [52]. Members of the *Chloroflexi* and deep-sea archaeal groups were dominant microorganisms in the surface sediment associated with the Iheya North hydrothermal field [20]. The taxonomic assignments of both 16S rRNA and metagenome revealed that bacteria belonging to *Deltaproteobacteria* showed great abundance in the sediment of the Tangyin hydrothermal field in the southern Okinawa Trough, which was about 20% of the total microbial population. Moreover, we also found a high abundance of active bacteria belonging to *Deltaproteobacteria* (average 30%) in the RNA sample. The differences in the abundances of *Delta*-, *Gammaproteobacteria*, and *Anaerolineae* using our 16S rRNA gene and metagenomic data may be attributed to the CTAB method for DNA extraction, which starts with a higher DNA volume and is more accurate. *Deltaproteobacteria* was reported to be the dominant bacterial group in several hydrothermal fields, such as the Guaymas Basin and the Southwest Indian Ridge, accounting for about 24% and 23.4%, respectively [13,56,57]. However, the proportion of *Deltaproteobacteria* was only 5.9% according to the 16S rRNA gene sequences retrieved from the metagenome in the hydrothermal fields of Juan de Fuca Ridge [58]. MAGs affiliated with *Desulfobacterota* and *Thermoproteota* were also abundant in our metagenome data. This implies that SRB may play an important role in the sediment of the Tangyin hydrothermal field. Furthermore, our previous study found that *Bacteroidetes* were predominant in the Tangyin hydrothermal field, which was consistent with our present results [18]. Previous reports have also indicated that, compared to bacterial communities in active chimneys, where *Epsilonproteobacteria* were dominated, bacterial communities in inactive chimneys and cooler sediments are not affected by hydrothermal circulation [59]. These results are consistent with the results in our study, in which the sampling site was about 1200 m away from the active vent site. Our results support the hypothesis that *Alpha*-, *Beta*-, *Delta*-, and *Gammaproteobacteria* prefer low-temperature habitats away from active hydrothermal regions [4].

Deep-sea hydrothermal vents are the main sources of CH_4_, sulfur, and metals in the ocean, and diverse microbial populations containing these metabolic pathways play important roles in carbon, sulfur, and iron cycles [60]. The CH_4_ concentrations at the Yonaguni Knoll IV field in Okinawa Trough are one to two orders of magnitude higher than those at unsedimented mid-oceanic ridge hydrothermal sites in the Pacific, Atlantic, and Indian Oceans [1], which is favorable for conducting detailed studies of microbial CH_4_ consumption in hydrothermal fields from Okinawa Trough. In addition, the hydrothermal fluid chemistry in the Iheya North field is highly influenced by the presence of thick terrigenous sediments in the trough basin and is characterized by high concentrations of CH_4_ and CO_2_ and a relatively low concentration of iron and molecular hydrogen [61]. The hydrothermal fluid inputs also have a remarkable impact on microbial community structures and functions in the deeper sediments of the peripheral area, 2 m from the vent site [21]. In the metagenome from the Tangyin hydrothermal field in the southern part of Okinawa Trough, we found a great number of genes involved in methane metabolism. The most abundant enzyme MetF was a key enzyme of methane metabolism and could be found in many methanogens, such as *Methanosarcina barkeri* [62], *Methanosarcina thermophila* [63], and *Methanothermobacter marburgensis* [64]. However, genes coding MetF in our metagenomic analyses were mainly affiliated with *Methylococcales* and *Methylococcaceae*. In addition, carbon fixation is also an essential metabolic process identified in deep-sea hydrothermal vents [46], and genes associated with carbon fixation affiliated with the CBB cycle and the Wood–Ljungdahl pathway were also identified in this study.

Notably, SRB dominated in the sediment sample of the Tangyin hydrothermal field both in terms of abundance (the abundance of reads mapped to MAGs containing genes associated with sulfate reduction) and diversity (the number of MAGs containing genes associated with sulfate reduction). Sulfate reduction is a globally important microbial process in the anoxic marine sediment, which could be detected in sulfate-rich zones as well as in sediment with low background concentrations of sulfate [65]. In this study, the process of sulfite reduction to sulfide was predominant, and a high abundance of genes, *dsrABC* (mediating dissimilatory sulfite reduction to sulfide) and *hydABDG* (mediating sulfur polysulfides reduction to sulfide), were observed. In addition, there were also many genes (*sat*, *aprAB*) that mediated sulfate to APS, APS reduction to sulfite, and sulfate to PAPS, respectively. Moreover, the complete dissimilatory sulfate reduction to the APS pathway was found in the MAGs of *Fermentibacterota* (containing *sat* gene), *Gemmatimonadota* (containing *dsrABC* and *sat* genes), and *Zixibacteria* (containing *dsrABC* and *sat* genes), and these bacteria had also been found in mesothermal sediments of the Guaymas Basin and Haima cold seep, indicating that they could adapt to marine environments with various geochemical features [66]. However, MAGs of these three bacterial groups did not contain genes related to high-temperature tolerance and dissimilatory sulfate reduction in previous studies [66]. Further investigations are required to confirm their capabilities in dissimilatory sulfate reduction. As for sulfide oxidation, Sqr was also abundant in the sediment of the Tangyin hydrothermal field, catalyzing sulfide to sulfur. Sqr has a strong affinity for high sulfide concentrations, implying that the sediment in the Tangyin hydrothermal field constitutes a high-sulfide environment [67]. In addition, *Bacteroidota* is a common microbial group in the hydrothermal sediments of the Guaymas Basin and Haima cold seep [68,69]. It is worth noting that two MAGs of *Bacteroidota*, which had complete sulfide oxidation and ferrous/ferric transporter metabolic pathways, were obtained in our study. It can be inferred that sulfate reduction and sulfur oxidation were key sulfur metabolisms in the Tangyin hydrothermal field, and SRB and SOB played important roles in the hydrothermal sediment environment.

Iron is one of the limiting nutrients in marine environments and hydrothermal fields are considered to be the main resources of iron [70]. Because it is such a limiting nutrient, marine microorganisms employ multiple strategies for obtaining Fe in various forms, including dissolved Fe, particulate Fe (minerals), and Fe tightly bound to organic complexes, such as siderophores, hemophores, and heme [71]. Vent-derived iron was previously thought to rapidly oxidize and precipitate around vents. However, organic matter can bind to and stabilize dissolved and particulate iron in hydrothermal plumes, facilitating its dispersion into the open ocean [70]. Previous studies mainly focused on sulfur and carbon-related anaerobic microorganisms in hydrothermal fields, but the important role of iron-related microorganisms in anaerobic sediments is ignored. In this study, diverse microorganisms (*Desulfobacterales*, *Deltaproteobacteria*, and *Campylobacterales*) and related genes (*exbB*, *feoB*, and *fbpC*) involved in the iron cycle were identified in the sediment sample of Tangyin hydrothermal field. Additionally, there was a relatively high abundance of bacteria belonging to *Zetaproteobacteria* associated with iron oxidation, which is considered the main driver of bio-mediated Fe^2+^ oxidation in hydrothermal vents and their surroundings. This shows that sulfur and iron metabolisms are essential for the Tangyin hydrothermal system. Genes involved in iron regulation and Fe^2+^ uptake were found in *Desulfobacterota, Gammaproteobacteria*, and *Chloroflexota*. It is noteworthy that genes related to iron oxidation or dissimilatory iron reduction were not identified in these organisms. However, we found a high abundance of iron gene regulators (*exbB*, *fur*, and *feoB*), commonly found in association with iron oxidation/reduction/transport-related genes [71,72,73]. A previous study identified two iron regulators adjacent to a gene encoding the iron reduction protein, suggesting a potential link between dissimilatory iron reduction and iron uptake [72,74,75]. Although the iron oxidation/reduction genes were not identified, the significant abundance of the ferric uptake gene (*feoB*) implies that bacteria in Tangyin utilize iron, particularly in the Fe^3+^ form, to cover their metabolic needs. Our results contribute valuable insights into enriching the diversity and metabolic functions of microorganisms in the Tangyin hydrothermal field and reveal their ecological function related to the carbon, sulfur, and iron cycles in this extreme environment.

## 5. Conclusions

The Tangyin hydrothermal vent is a newly found hydrothermal setting in the southern part of the Okinawa Trough. Previous studies have primarily concentrated on the geological features of this area, and there is limited knowledge regarding microbial composition and ecological function. In this study, a surficial sediment sample located 1.2 km away from the active vent was collected for 16S rRNA gene and metagenomic analyses. The prokaryotic communities in the sediment can fix carbon, uptake iron, and perform dissimilatory sulfate reduction. These processes are common in our data and other similar hydrothermal settings. The lack of methanogens and dominance of SRB can be attributed to the fact that the sample was collected on a cooler site, which is not under the immediate hydrothermal influence in the area. Comparative genomic analyses of representative groups *Bathyarchaeia* and *Thioglobaceae* from the Tangyin hydrothermal field reveal both similar and distinct metabolic potentials associated with carbon, sulfur, and iron metabolisms. In future studies, detailed 16S rRNA gene and metagenomic investigations at Tangyin sites with different temperature and geochemical profiles are necessary to describe the genomic potential and metabolic activities of the in situ sedimental prokaryotic community. Additionally, future research efforts should focus on the isolation and cultivation of these microorganisms to explore their metabolic mechanisms related to carbon, sulfur, and iron cycles in deep-sea hydrothermal fields.

## Data Availability

The 16S rRNA gene and metagenome data were deposited in NODE under the Project IDs OEP004983 and OEP004987, respectively.

## Supplementary Materials

The following supporting information can be downloaded at https://www.mdpi.com/article/10.3390/microorganisms12030517/s1, Table S1: Quality score and GTDB taxonomy of 34 MAGs retrieved from the Tangyin hydrothermal vent and 17 reference genomes; Table S2: Quality score and GTDB taxonomy of reference MAGs.
