# Supplementary material for: Deciphering Microbial Communities and Distinct Metabolic Pathways in the Tangyin Hydrothermal Fields of Okinawa Trough through Metagenomic and Genomic Analyses
Source: Microorganisms. 2024 Mar 4;12(3):517. doi: 10.3390/microorganisms12030517 (PMC10974373; doi:10.3390/microorganisms12030517)
Supplement: Supplementary file 1 [file microorganisms-12-00517-s001.zip › microorganisms-2867290-supplementary.pdf]

# *Supplementary Materia*

## Supplementary Tables

**Table S1.** Quality score and GTDB taxonomy of 34 MAGs retrieved from the Tangyin hydrothermal vent and 17 reference genomes.

| Genome | Completeness | Contamination | Taxonomy |
|---|---|---|---|
| MAG16 | 94.20529801 | 0.220750552 | d__Bacteria; p__Proteobacteria; c__Gammaproteobacteria; o__PS1; f__Thioglobaceae; g__; s__ |
| MAG29 | 89.73160796 | 2.803738318 | d__Bacteria; p__Thermoproteota; c__Bathyarchaeia; o__TCS64; f__TCS64; g__GCA-2726865; s__ |
| MAG19 | 88.85304659 | 1.612903226 | d__Bacteria; p__Desulfobacterota; c__Desulfobacteria; o__Desulfobacterales; f__SURF-7; g__; s__ |
| MAG32 | 87.06896552 | 3.761755486 | d__Bacteria; p__Chloroflexota; c__Anaerolineae; o__Anaerolineales; f__UBA11858; g__UBA11858; s__ |
| MAG4 | 83.82838284 | 0 | d__Bacteria; p__Chloroflexota; c__Dehalococcoidia; o__SZUA-161; f__SZUA-161; g__; s__ |
| MAG23 | 81.25448029 | 0.896057348 | d__Bacteria; p__Desulfobacterota; c__Desulfobacteria; o__Desulfatiglandales; f__; g__; s__ |
| MAG15 | 81.17913832 | 1.636904762 | d__Bacteria; p__Desulfobacterota; c__Desulfobulbia; o__Desulfobulbales; f__BM004; g__BM004; s__ |
| MAG12 | 78.48358357 | 2.248941319 | d__Bacteria; p__Proteobacteria; c__Gammaproteobacteria; o__Thiohalobacterales; f__DSM-19610; g__Thiogranum; s__ |
| MAG6 | 76.79723502 | 1.290322581 | d__Bacteria; p__Desulfobacterota; c__Desulfobacteria; o__Desulfobacterales; f__SURF-7; g__; s__ |
| MAG24 | 73.87096774 | 1.612903226 | d__Bacteria; p__Desulfobacterota; c__Desulfobacteria; o__Desulfobacterales; f__BuS5; g__; s__ |
| MAG30 | 73.34833527 | 0.571041425 | d__Bacteria; p__Proteobacteria; c__Gammaproteobacteria; o__Chromatiales; f__Sedimenticolaceae; g__; s__ |
| MAG9 | 70.90919701 | 1.680672269 | d__Bacteria; p__Desulfobacterota; c__; o__; f__; g__; s__ |
| MAG13 | 67.81003584 | 1.095750128 | d__Bacteria; p__Desulfobacterota; c__Desulfobacteria; o__C00003060; f__C00003060; g__; s__ |
| MAG18 | 66.65566557 | 2.090209021 | d__Bacteria; p__Chloroflexota; c__Dehalococcoidia; o__; f__; g__; s__ |
| MAG10 | 66.25759459 | 1.232394366 | d__Bacteria; p__Proteobacteria; c__Gammaproteobacteria; o__UBA4486; f__UBA4486; g__SMWN01; s__ |
| MAG27 | 66.06024289 | 1.681048258 | d__Bacteria; p__Bacteroidota; c__Bacteroidia; o__Flavobacteriales; f__Flavobacteriaceae; g__QNYL01; s__ |
| MAG17 | 65.86844528 | 2.697302697 | d__Bacteria; p__Fermentibacterota; c__Fermentibacteria; o__Fermentibacterales; f__Fermentibacteraceae; g__Aegiribacteria; s__ |
| MAG33 | 65.37137114 | 4.651080827 | d__Bacteria; p__Bacteroidota; c__Bacteroidia; o__Cytophagales; f__Cyclobacteriaceae; g__; s__ |
| MAG21 | 63.31738437 | 0 | d__Bacteria; p__Bacteroidota; c__Bacteroidia; o__Flavobacteriales; f__Flavobacteriaceae; g__QNYL01; s__ |
| MAG22 | 63.05948953 | 1.648351648 | d__Bacteria; p__Eisenbacteria; c__; o__; f__; g__; s__ |
| MAG31 | 62.53779289 | 1.785714286 | d__Bacteria; p__Desulfobacterota; c__Desulfobulbia; o__Desulfobulbales; f__Desulfobulbaceae; g__Electrothrix; s__ |
| MAG8 | 61.57706093 | 0 | d__Bacteria; p__Desulfobacterota; c__Desulfobacteria; o__Desulfobacterales; f__UBA5852; g__; s__ |
| MAG25 | 60.47654596 | 1.32387462 | d__Bacteria; p__Proteobacteria; c__Gammaproteobacteria; o__Woeseiales; f__Woeseiaceae; g__UBA1847; s__ |
| MAG14 | 60.00921659 | 0.921658986 | d__Bacteria; p__Desulfobacterota; c__Desulfobacteria; o__Desulfobacterales; f__BuS5; g__UBA11574; s__ |
| MAG28 | 58.62068966 | 0 | d__Bacteria; p__Proteobacteria; c__Gammaproteobacteria; o__UBA10353; f__LS-SOB; g__; s__ |
| MAG3 | 57.24471607 | 5.238878768 | d__Bacteria; p__Gemmatimonadota; c__Gemmatimonadetes; o__Gemmatimonadales; f__GWC2-71-9; g__; s__ |
| MAG1 | 55.85909418 | 2.803738318 | d__Bacteria; p__Thermoproteota; c__Bathyarchaeia; o__TCS64; f__TCS64; g__RBG-16-57-9; s__ |
| MAG20 | 54.9489726 | 1.098901099 | d__Bacteria; p__Zixibacteria; c__MSB-5A5; o__; f__; g__; s__ |
| MAG2 | 54.4488656 | 4.000603865 | d__Bacteria; p__Proteobacteria; c__Gammaproteobacteria; o__Xanthomonadales; f__SZUA-36; g__; s__ |
| MAG7 | 52.9109063 | 2.580645161 | d__Bacteria; p__Desulfobacterota; c__Desulfobacteria; o__Desulfobacterales; f__BuS5; g__S5133MH16; s__ |
| MAG34 | 52.87114846 | 2.315592904 | d__Bacteria; p__Proteobacteria; c__Zetaproteobacteria; o__Mariprofundales; f__Mariprofundaceae; g__; s__ |
| MAG11 | 52.50332821 | 2.424475166 | d__Bacteria; p__Desulfobacterota; c__Desulfobacteria; o__Desulfobacterales; f__BuS5; g__; s__ |
| MAG26 | 52.36303123 | 0.537634409 | d__Bacteria; p__Desulfobacterota; c__Desulfobacteria; |

| | | | |
|---|---|---|---|
| | | | o__Desulfobacterales; f__SG8-13; g__; s__ |
| | | | (Continued) |
| MAG5 | 51.96523054 | 0 | d__Bacteria; p__Desulfobacterota; c__Desulfobulbia; o__Desulfobulbales; f__Desulfobulbaceae; g__QNYF01; s__ |
| GCA_000307955.1 | 100 | 0 | d__Bacteria; p__Desulfobacterota; c__Desulfovibrionia; o__Desulfovibrionales; f__Desulfovibrionaceae; g__Solidesulfovibrio; s__Solidesulfovibrio magneticus_A |
| GCA_001797445.1 | 94.96 | 1.68 | d__Bacteria; p__Desulfobacterota_E; c__Deferrimicrobia; o__Deferrimicrobiales; f__Deferrimicrobiaceae; g__Deferrimicrobium; s__Deferrimicrobium sp001797445 |
| GCA_001797465.1 | 94.84 | 2.26 | d__Bacteria; p__Desulfobacterota_F; c__GWC2-55-46; o__GWC2-55-46; f__GWC2-55-46; g__GWB2-55-19; s__GWB2-55-19 sp001797465 |
| GCA_002030045.1 | 95.05 | 2.2 | d__Bacteria; p__Fermentibacterota; c__Fermentibacteria; o__Fermentibacterales; f__Fermentibacteraceae; g__Aegiribacteria; s__Aegiribacteria sp002030045 |
| GCA_014728175.1 | 96.15 | 1.28 | d__Bacteria; p__Fermentibacterota; c__Fermentibacteria; o__Fermentibacterales; f__Fermentibacteraceae; g__WJMA01; s__WJMA01 sp014728175 |
| GCA_005893265.1 | 92.66 | 1.1 | d__Bacteria; p__Eisenbacteria; c__RBG-16-71-46; o__SZUA-252; f__SZUA-252; g__WS-7; s__WS-7 sp005893265 |
| GCA_001780165.1 | 98.9 | 0 | d__Bacteria; p__Eisenbacteria; c__RBG-16-71-46; o__RBG-16-71-46; f__RBG-16-71-46; g__RBG-16-71-46; s__RBG-16-71-46 sp001780165 |
| GCA_016928395.1 | 91.16 | 3.3 | d__Bacteria; p__Fermentibacterota; c__JAFGKV01; o__JAFGKV01; f__JAFGKV01; g__JAFGKV01; s__JAFGKV01 sp016928395 |
| GCA_016936235.1 | 92.31 | 2.2 | d__Bacteria; p__Fermentibacterota; c__Fermentibacteria; o__Fermentibacterales; f__Fermentibacteraceae; g__Aegiribacteria; s__Aegiribacteria sp016936235 |
| GCA_003641425.1 | 93.1 | 2.5 | d__Bacteria; p__Zixibacteria; c__MSB-5A5; o__GN15; f__PGXB01; g__PGXB01; s__PGXB01 sp003641425 |
| GCA_003599535.1 | 91.21 | 1.1 | d__Bacteria; p__AABM5-125-24; c__B3-LCP; o__B3-LCP; f__B3-LCP; g__SURF-9; s__SURF-9 sp003599535 |
| GCA_001567485.1 | 97.27 | 0 | d__Bacteria; p__Chloroflexota; c__Anaerolineae; o__Aggregatilineales; f__Phototrophicaceae; g__OLB13; s__OLB13 sp001567485 |
| GCF_000526415.1 | 97.58 | 0 | d__Bacteria; p__Chloroflexota; c__Chloroflexia; o__Chloroflexales; f__Herpetosiphonaceae; g__JKG1; s__JKG1 sp000526415 |
| GCF_000193795.1 | 99.7 | 0 | d__Bacteria; p__Pseudomonadota; c__Gammaproteobacteria; o__Burkholderiales; f__Neisseriaceae; g__Neisseria; s__Neisseria lactamica_A |
| GCA_000016325.1 | 99.7 | 0.1 | d__Bacteria; p__Pseudomonadota; c__Gammaproteobacteria; o__Enterobacterales; f__Enterobacteriaceae; g__Lelliottia; s__Lelliottia sp000016325 |
| GCA_000263195.1 | 100 | 0.3 | d__Bacteria; p__Bacteroidota; c__Bacteroidia; o__Cytophagales; f__Spirosomaceae; g__Emticicia; s__Emticicia oligotrophica |
| GCA_000434235.1 | 98.1 | 0.96 | d__Bacteria; p__Bacteroidota; c__Bacteroidia; o__Bacteroidales; f__Rikenellaceae; g__Alistipes; s__Alistipes avistercoris |
| GCA_018396865.1 | 94.86 | 0 | d__Archaea; p__Thermoproteota; c__Bathyarchaeia; o__TCS64; f__TCS64; g__JAGTRB01; s__JAGTRB01 sp018396865 |
| GCA_000145985.1 | 100 | 0 | d__Archaea; p__Thermoproteota; c__Thermoprotei_A; o__Sulfolobales; f__Ignisphaeraceae; g__Ignisphaera; s__Ignisphaera aggregans |

**Table S2.** Quality score and GTDB taxonomy of reference MAGs.

| Genome | Completeness | Contamination | Taxonomy |
|---|---|---|---|
| GCA_001593855.1 | 74.3 | 1.32 | d__Archaea; p__Thermoproteota; c__Bathyarchaeia; o__B25; f__B25; g__B25; s__B25 sp001593855 |
| GCA_002255025.1 | 73.83 | 0.93 | d__Archaea; p__Thermoproteota; c__Bathyarchaeia; o__ex4484-135; f__ex4484-135; g__ex4484-135; s__ex4484-135 sp002255025 |
| GCA_003661975.1 | 83.64 | 6.07 | d__Archaea; p__Thermoproteota; c__Bathyarchaeia; o__B26-1; f__B26-1; g__B63; s__B63 sp003661975 |
| GCA_001273345.1 | 50.34 | 9.22 | d__Archaea; p__ Bathyarchaeota; s__ SG8-32-1 |
| GCA_002726865.1 | 92.99 | 3.31 | d__Archaea; p__Thermoproteota; c__Bathyarchaeia; o__TCS64; f__TCS64; g__GCA-2726865; s__GCA- |

2726865 sp002726865

| | | | |
|---|---|---|---|
| (Continued) | | | |
| GCA_002010925.1 | 77.28 | 1.25 | d__Archaea; p__Thermoproteota; c__Bathyarchaeia; o__B26-1; f__UBA233; g__UBA233; s__UBA233 sp002010925 |
| GCA_004376295.1 | 97.82 | 1.87 | d__Archaea; p__Thermoproteota; c__Bathyarchaeia; o__B26-1; f__BA1; g__SOJZ01; s__SOJZ01 sp004376295 |
| GCA_001768965.1 | 72.26 | 1.94 | d__Archaea; p__Thermoproteota; c__Bathyarchaeia; o__40CM-2-53-6; f__FEN-987; g__FEN-987; s__FEN-987 sp001768965 |
| GCA_003978185.1 | 60.21 | 0.7 | d__Bacteria; p__Pseudomonadota; c__Gammaproteobacteria; o__PS1; f__Thioglobaceae; g__Thioglobus_C; s__Thioglobus_C sp003978185 |
| GCA_012964205.1 | 90.02 | 4.25 | d__Bacteria; p__Pseudomonadota; c__Gammaproteobacteria; o__PS1; f__Thioglobaceae; g__Thioglobus_A; s__Thioglobus_A sp012963715 |
| GCA_008080915.1 | 82.42 | 0 | d__Bacteria; p__Pseudomonadota; c__Gammaproteobacteria; o__PS1; f__Thioglobaceae; g__VMDI01; s__VMDI01 sp008080915 |
| GCA_001628345.1 | 86.6 | 3.53 | d__Bacteria; p__Pseudomonadota; c__Gammaproteobacteria; o__PS1; f__Thioglobaceae; g__Pseudothioglobus; s__Pseudothioglobus sp001628405 |
| GCA_014384345.1 | 97.35 | 0 | d__Bacteria; p__Pseudomonadota; c__Gammaproteobacteria; o__PS1; f__Thioglobaceae; g__Thioglobus_A; s__Thioglobus_A pontius |
| GCA_018645225.1 | 95.81 | 0 | d__Bacteria; p__Pseudomonadota; c__Gammaproteobacteria; o__PS1; f__Thioglobaceae; g__SUP05; s__SUP05 sp000205985 |
| GCA_018697175.1 | 78.57 | 5.68 | d__Bacteria; p__Pseudomonadota; c__Gammaproteobacteria; o__PS1; f__Thioglobaceae; g__SUP05; s__SUP05 sp000205985 |
